# Multifunctional surface designed by nanocomposite coating of polytetrafluoroethylene and TiO_2_ photocatalyst: self-cleaning and superhydrophobicity

**DOI:** 10.1038/s41598-017-14058-9

**Published:** 2017-10-19

**Authors:** Takashi Kamegawa, Koichi Irikawa, Hiromi Yamashita

**Affiliations:** 1 Division of Materials and Manufacturing Science, Graduate School of Engineering, Osaka University, 2-1 Yamadaoka, Suita, Osaka, 565-0871 Japan; 2 NanoSquare Research Institute, Osaka Prefecture University, 1-2 Gakuencho, Nakaku, Sakai, Osaka, 599-8570 Japan; 3 Unit of Elements Strategy Initiative for Catalysts and Batteries (ESICB), Kyoto University, Katsura, Kyoto, 615-8510 Japan

## Abstract

Multifunctional surface, having both a superhydrophobic property and a photocatalytic self-cleaning property, was designed through a nanocomposite coating of polytetrafluoroethylene (PTFE) and TiO_2_ photocatalyst onto a flat quartz glass with a precise structural controlling by applying a radio frequency magnetron sputtering deposition technique. Systematic water contact angle measurements were carried out in relation to the controlling of the surface structure such as size, height and others. Surface wettability gradually changes from Wenzel state to Cassie-Baxter state by controlling of the surface structure, resulting in a well water repellent behavior. Under irradiation of UV light, nanocomposite coating with a desired surface structure and composition realized an adequate photocatalytic self-cleaning property for keeping a clean surface and inducing unique surface wettability changes.

## Introduction

The surface wettability of various materials is an important subject that has a great interest for long periods^1–4^. Surface wetting behavior is possible to be roughly classified into two categories, *i.e*., hydrophilic state and hydrophobic state, depending on the behavior of water droplet on the surface of target materials. Especially, unusual surface wetting behavior observed in superhydrophilic surface (water contact angle (*θ*): less than 5°) and superhydrophobic surface (*θ*: higher than 150°) has attracted substantial interest in technological applications^4–7^. In the case of superhydrophilic surface, water droplet gets wet and rapidly spreads to the entire surface with a high uniformity. Such function is already commercialized as coatings for door mirrors of cars, outside panels of buildings and others. Intriguing phenomenon, *i.e*., photoinduced superhydrophilicity, is also observed in TiO_2_ surface and applied in the design of multifunctional superhydrophilic surface with a simultaneous use of TiO_2_ photocatalysis for self-cleaning^8–12^. TiO_2_ is a well-known photocatalyst for removal of diluted organic molecules in air and water, which is also being studied in the energy related issues^13–22^. As an opposite wetting behavior, water droplet with spherical shape is also observed in the case of superhydrophobic surface, which is extremely repellent for water and other substances^23–25^. This surface is observed in natural creatures such as leaves of plant, legs of water strider, wings of butterfly and others^26–29^. Such function is also used to solve a lot of undesired things such as snow adhering, corrosion and others. The necessary requirements of this surface are a low surface energy of material as well as a high roughness with micro- or nano-structured surface architecture. Water contact angle is ca. 120° on a flat fluorine polymer surface with a quite low surface energy. Diverse techniques for modification and construction of desired surface architecture are indispensable in agreement with necessary requirements. The model surface with pillars, wires and needles was designed by some techniques (*e.g*. lithography, etching and others) in previous works. Polymers, block-copolymers and various chemicals are adopted in preparation processes. Not only does the modified surface show simple superhydrophobicity, but it also has multifunctional properties. Surface wetting behavior is controllable by external stimuli such as temperature, pH, and others, which attain the reversible switching of surface wettability between superhydrophobic and superhydrophilic states^30–35^.

**Figure 1:**
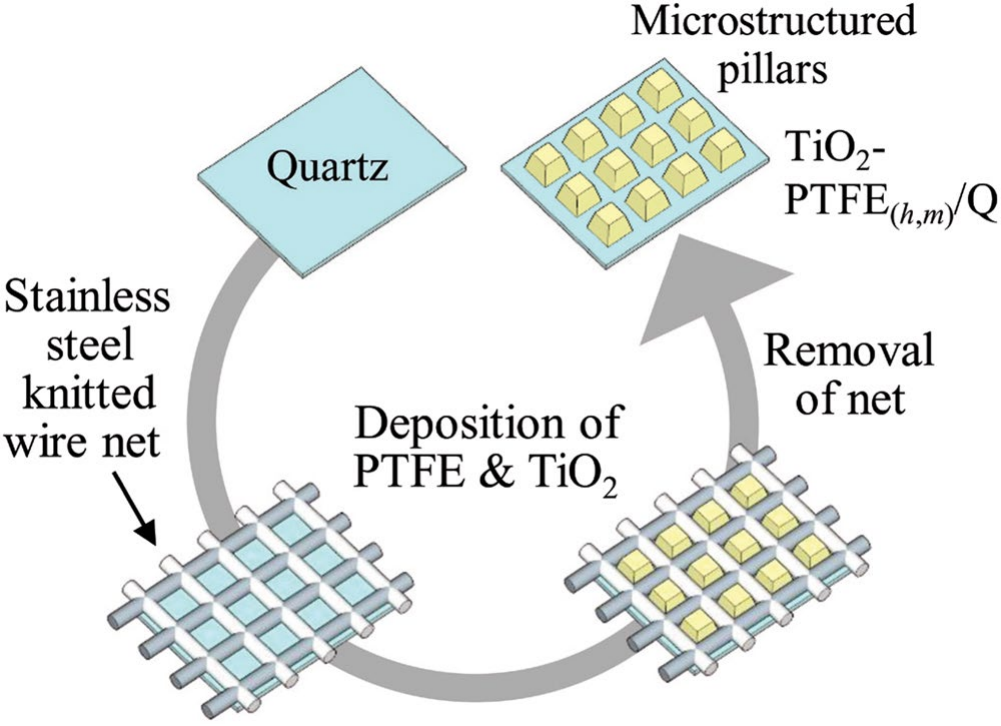
Illustration of the procedures for design of periodically structured TiO_2_-PTFE composite sample.

Coating technologies are strongly related to dramatic changes of surface wetting behavior and thus continuously investigated by many researchers^36–41^. Composite coatings are also effective techniques for accumulating multiple functions on substrate surface. However, there are some limitations for accumulation of fascinating functions in relation to the incompatibility of inherent property of materials. For instance, the combination of hydrophobic materials and TiO_2_ has not yet received a full investigation due to the photoinduced superhydrophilicity of TiO_2_^39–41^. The selection and precise compositional control of key building blocks in composite materials is indispensable for accumulating desired functions. In the present work, systematic surface wettability investigations were firstly carried out through the design and water contact angle measurement of the polytetrafluoroethylene (PTFE)-coated quartz glass with a well-defined pillar structure, aimed at the clarifying the required parameters for realizing a superhydrophobic state. A stainless steel knitted wire net with a different size of square openings was adopted as a simple method for making pillars on a flat quartz glass (Fig. 1). By the nanocomposite coating of PTFE and TiO_2_ photocatalyst, multifunctional surface was then realized with an adequate superhydrophobic property and photocatalytic self-cleaning property for keeping a clean surface and inducing unique surface wettability changes.

## Results and Discussion

### Surface wettability of periodically structured fluoropolymer deposited samples

**Figure 2:**
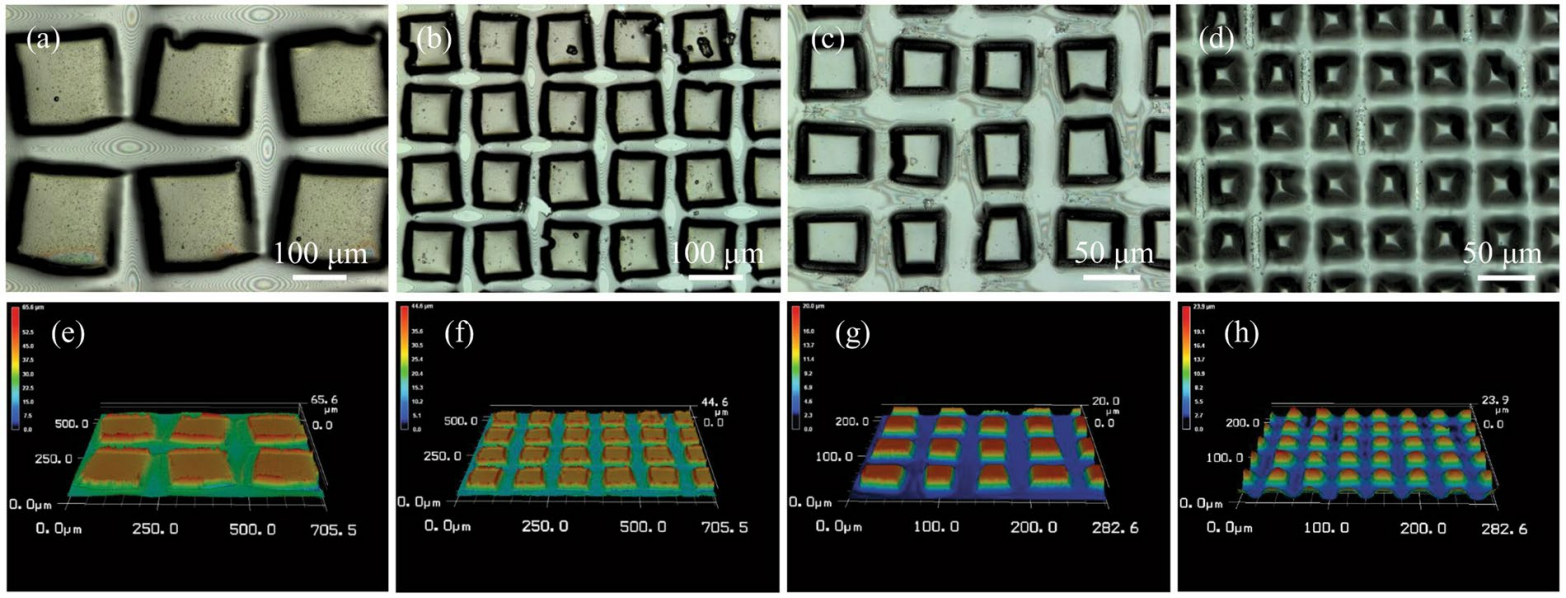
(**a–d**) Optical images and (**e–h**) 3D analytical laser microscope images of PTFE_(*15*,*m*)_/Q (*m* = (**a,e**) 100, (**b,f**) 200, (**c,g**) 400, and (**d,h**) 635).

Figure 2 shows the optical and laser scanning microscope image of PTFE deposited samples prepared on a flat quartz substrate (PTFE_(*h*,*m*)_/Q) through a stainless steel knitted wire net with a different size of square openings (*m* = 100, 200, 400 and 635). Microstructured PTFE pillars were directly constructed on a quartz substrate with almost same pillars height (*h* = ca. 15 μm) by controlling of the deposition time. Microstructured PTFE pillars reflect a size of square openings in wire net. The entire surface of quartz substrate was also covered by microstructured PTFE pillars. The three dimensional analyses indicated that the PTFE pillars have truncated square pyramid structure. As shown in Supplementary Figure 1, PTFE_(*h*,*400*)_/Q (*h* = ca. 3, 10, 18 and 40 μm) shows gradual decreases in the flat top area of pillars depending on the PTFE deposition time, although each sample was prepared by using a same size of wire net (*m* = 400). The gradual decreases in the size of square openings due to the accumulation of deposited PTFE on knitted wire surface results in the structural changes in each sample. Deposited PTFE on a quartz substrate exhibited FT-IR peaks at ca. 1725, 1250 and 740 cm^−1^ attributed to the existence of CF = CF and CF_*n*_ (*n* = 1–3) groups^42,43^. The structure of PTFE was retained during the deposition on quartz substrate using an argon plasma.

**Figure 3:**
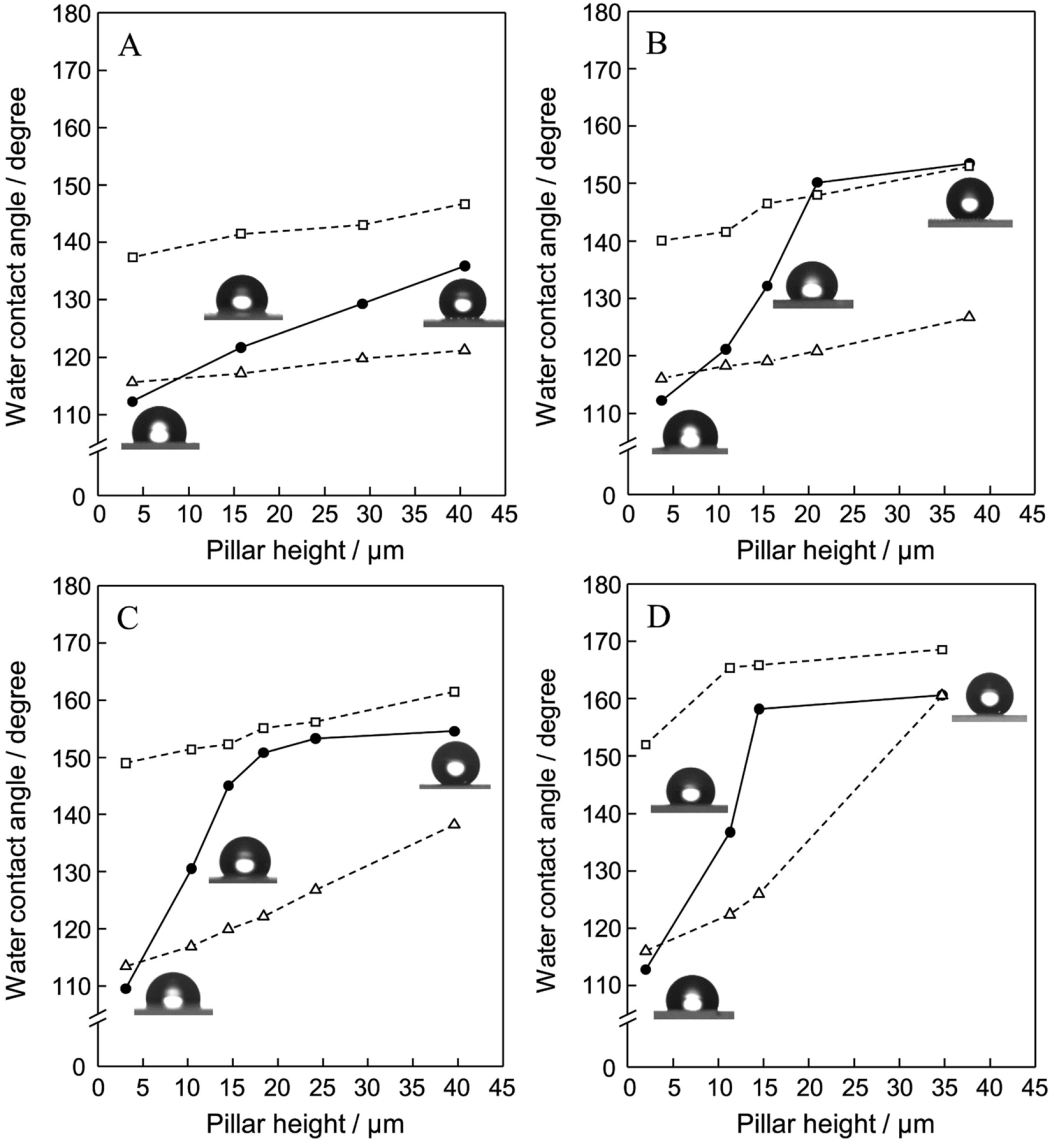
Relationships between the pillar height (*h*) and water contact angle (CA) on PTFE_(*h*,*m*)_/Q (*m* = (**A**) 100, (**B**) 200, (**C**) 400, and (**D**) 635). Dotted line shows the calculated value of CA by using Wenzel equation (open triangle) and Cassie-Baxter equation (open square). Inset of this figure shows the photographic images of water droplet on each sample.

The water contact angle measurements were carried out to evaluate the surface wetting properties of PTFE_*(h*,*m)*_/Q with a different pillar height. As shown in Fig. 3, depending on the pillar height, the water contact angle gradually increased from ca. 110° to 160°. The dotted line in Fig. 3 indicates the predictions of water contact angle calculated by the Wenzel equation (Eq. (1)) and Cassie-Baxter equation (Eq. (2)), respectively. Experimentally obtained water contact angle values are mainly located within two predicted lines. The theoretical values predicted by Cassie-Baxter equation overestimate the water contact angle, which may be due to the truncated square pyramid structure of pillars in each sample. Although PTFE has low surface energy, the water contact angle on a flat PTFE plate is only ca. 116°. Due to the surface property of quartz substrate, the water contact angle become slightly small on PTFE_(*h*,*m*)_/Q with low pillar height. Superhydrophobic state was achieved by construction of PTFE pillars height over ca. 20 μm. The air pockets between the asperities, which were constructed by pillars on substrate surface, play important roles for realizing a superhydrophobicity. Inset of Fig. 3 shows the photographic images of water droplet on each sample. The spherical water droplet was clearly observed on samples with desired surface architecture. On these points, the microstructured pillar size was also strictly affect to the changes of surface wettability. The higher water contact angle was attained on samples having more fine pillars with a truncated square pyramid structure. In this evaluation, PTFE_(*h*,*100*)_/Q have relatively large top area of pillars, which are difficult to attain the superhydrophobicity even after changing the pillar height below ca. 40 μm. The top area of pillars and the distance between the other adjacent pillars are also depended on the size of square openings in wire net. The ratio of pillar height (*h*) and distance (*d*) shows good relations with the water contact angle on each sample (Supplementary Figure 2). Distance from one pillar to another was measured at many different points on sample surface. An average value was adopted for calculation of ratio (*h/d*). Superhydrophobicity was observed on samples over the ratio of *h*/*d* = ca. 0.5. The construction of artificially designed surface solve a design of superhydrophobic surface on a flat substrate.

**Figure 4:**
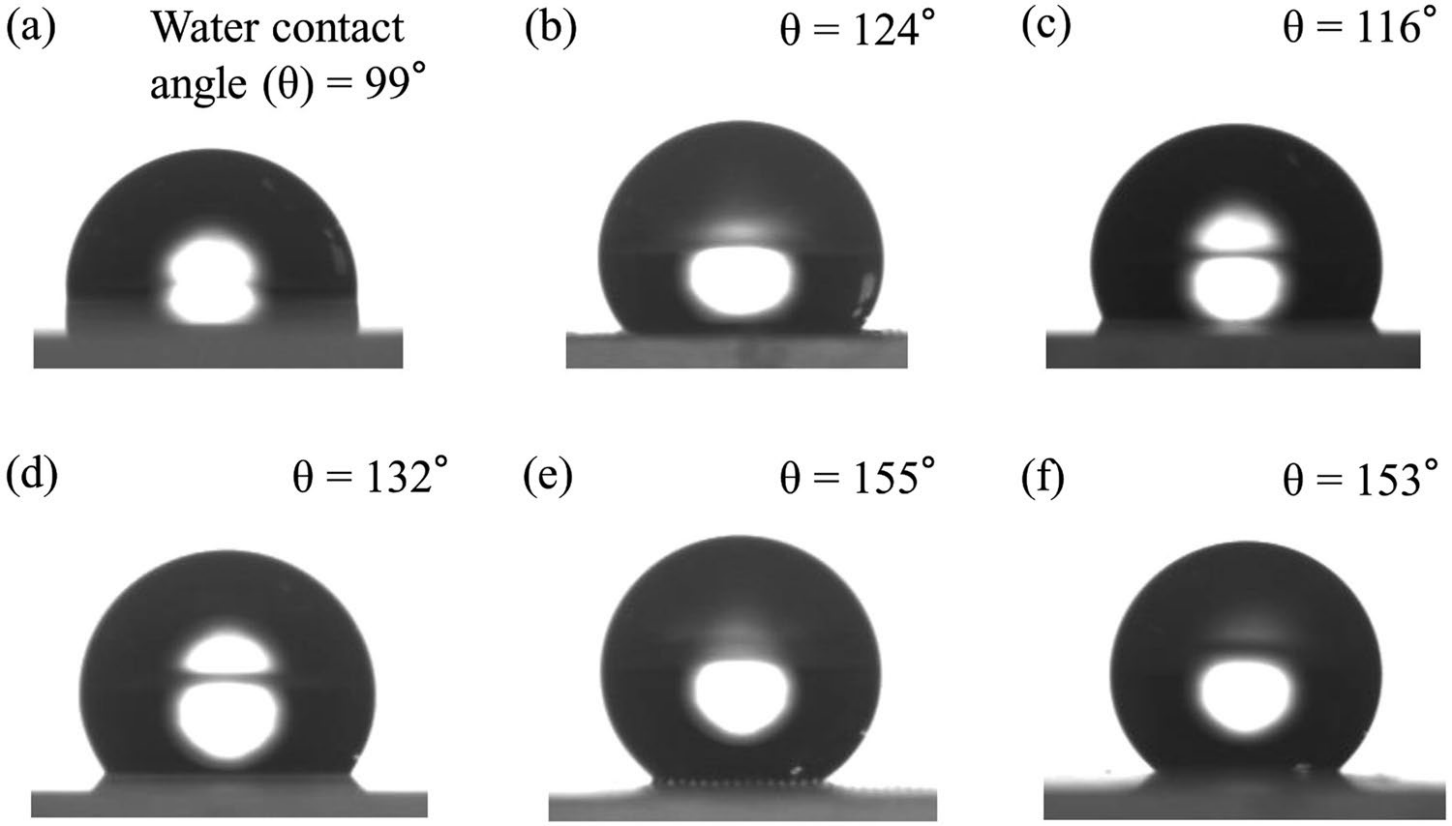
Photographic images of water dropped on sample surface ((**a**) PCTFE/Q, (**b**) FEP/Q, (**c**) PTFE/Q, (**d**) PCTFE_*(25,400)*_/Q, (**e**) FEP_(*25*,*400*)_/Q, and (**f**) PTFE_(*25*,*400*)_/Q).

The deposition of fluoropolymers such as PCTFE and FEP on a flat quartz substrate through a stainless steel knitted wire net (400 mesh) was also carried out by using a same method. The height of pillars was adjusted to ca. 25 μm. These polymers are well-known, commercialized, and used in the various fields. As shown in Supplementary Figure 3, microstructured pillars were successfully deposited on substrate surface, which were quite similar to those of PTFE_(*25*,*400*)_/Q. Photographic images of water droplet on these samples in the absence and presence of microstructured pillar structure were shown in Fig. 4. The water contact angle on a flat samples was determined to be ca. 99° (PCTFE/Q), 124° (FEP/Q) and 116° (PTFE/Q), respectively. The quite higher water contact angle was observed in the samples with microstructured pillar structure. The water contact angle follows the order of ca. 155° (FEP_*(25*,*400*)_/Q) > 153° (PTFE_(*25*,*400*)_/Q) > 132° (PCTFE_*(25*,*400)*_/Q). The opposite order was clearly observed in the surface energy of these fluoropolymers^44,45^. The differences in the monomer structure of these fluoropolymers directly affect to the water contact angle. FEP having a lower surface energy than PTFE and PCTFE exhibited a good water repellent property.

### Surface property of periodically structured TiO_2_-PTFE composite sample

**Figure 5:**
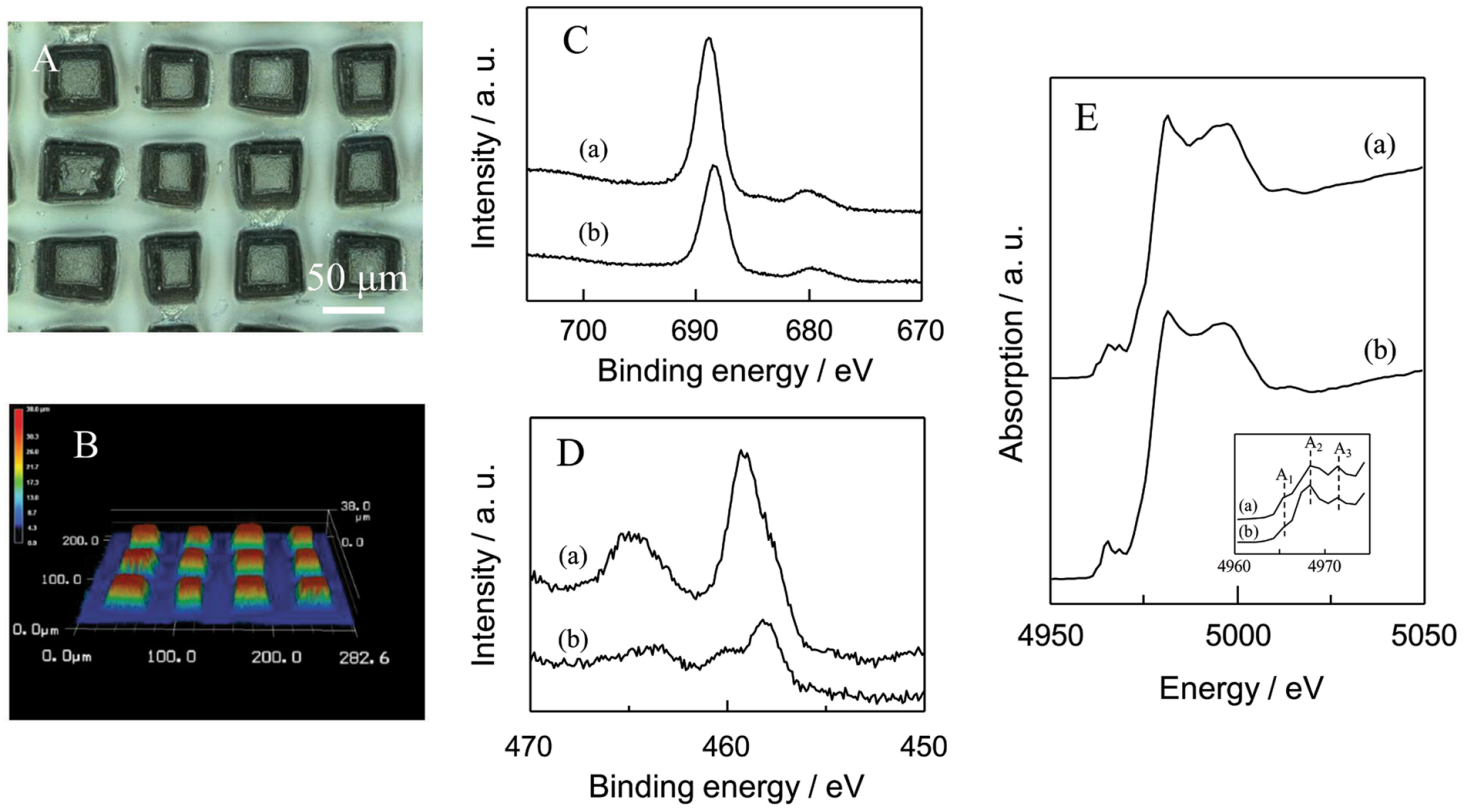
(**A**) Optical microscope images and (**B**) 3D analytical laser microscope images of TiO_2_-PTFE_(*25*,*400*)_/Q, (**C**) F_1s_ XPS spectra ((a) PTFE/Q and (b) TiO_2_-PTFE_*(25,400)*_/Q), (**D**) Ti_2p_ XPS spectra ((a) TiO_2_ (anatase) and (b) TiO_2_-PTFE_(*25*,*400*)_/Q), and (**E**) Ti K-edge X-ray absorption near edge structure spectra ((a) TiO_2_ (anatase) and (b) TiO_2_-PTFE_*(25*,*400)*_/Q).

To accumulate the specific functions on a flat quartz glass, the combination of the most often-used fluoropolymer (PTFE) and TiO_2_ photocatalyst was investigated in the design of superhydrophobic surface with self-cleaning property. The gradual loss of surface superhydrophobicity is caused by the unavoidable issues, such as the adhesion and accumulation of organic contaminants in air. This property is irreversibly disappeared without the removal of organic contaminants by a meticulous cleaning. In the decomposition of organic contaminants in air and water, TiO_2_ photocatalyst is effective and able to accomplish a purpose by only uses of clean light energy as an external stimulus. Various organic contaminants accumulated on TiO_2_ surface are oxidized finally into CO_2_ and H_2_O. However, TiO_2_ surface shows superhydrophilicity after irradiation of UV light. On this point, suitable technique is indispensable to advance the combination of hydrophobic material and TiO_2_. The combination of chemically inert PTFE and TiO_2_ as well as the shape controlling was carried out by the simultaneous deposition of two components through a stainless steel knitted wire net (400 mesh). Figure 5(A,B) shows optical and laser scanning microscope image of simultaneously PTFE and TiO_2_ deposited sample on a flat quartz substrate (TiO_2_-PTFE_(25,*400*)_/Q). The formation of microstructured pillars (height: ca. 25 μm) was clearly observed in these images. In the F_1s_ and Ti_2p_ XPS analyses (Fig. 5(C,D)), both peaks due to the existence of the fluorine in PTFE and titanium in TiO_2_ were observed in the region from 680 to 700 eV and 450 to 470 eV, respectively^41,42^. The crystalline structure of TiO_2_ in TiO_2_-PTFE composite sample was also investigated by XAFS measurement. Figure 5(E) shows the results of the X-ray absorption near edge structure (XANES) spectrum measurements. TiO_2_ (anatase) shows characteristic pre-edge peaks attributed to the transitions from the 1 s core level of Ti atom to three different kinds of orbitals (1t_1g_, 2t_2g_, and 3e_g_) of TiO_2_ with octahedral geometry^46–49^. TiO_2_-PTFE composite sample exhibited well-defined pre-edge peaks in the same position of TiO_2_ (anatase), indicating that this sample contains TiO_2_ with anatase structure. The concentration of TiO_2_ in the PTFE matrix was ca. 5%.

**Figure 6:**
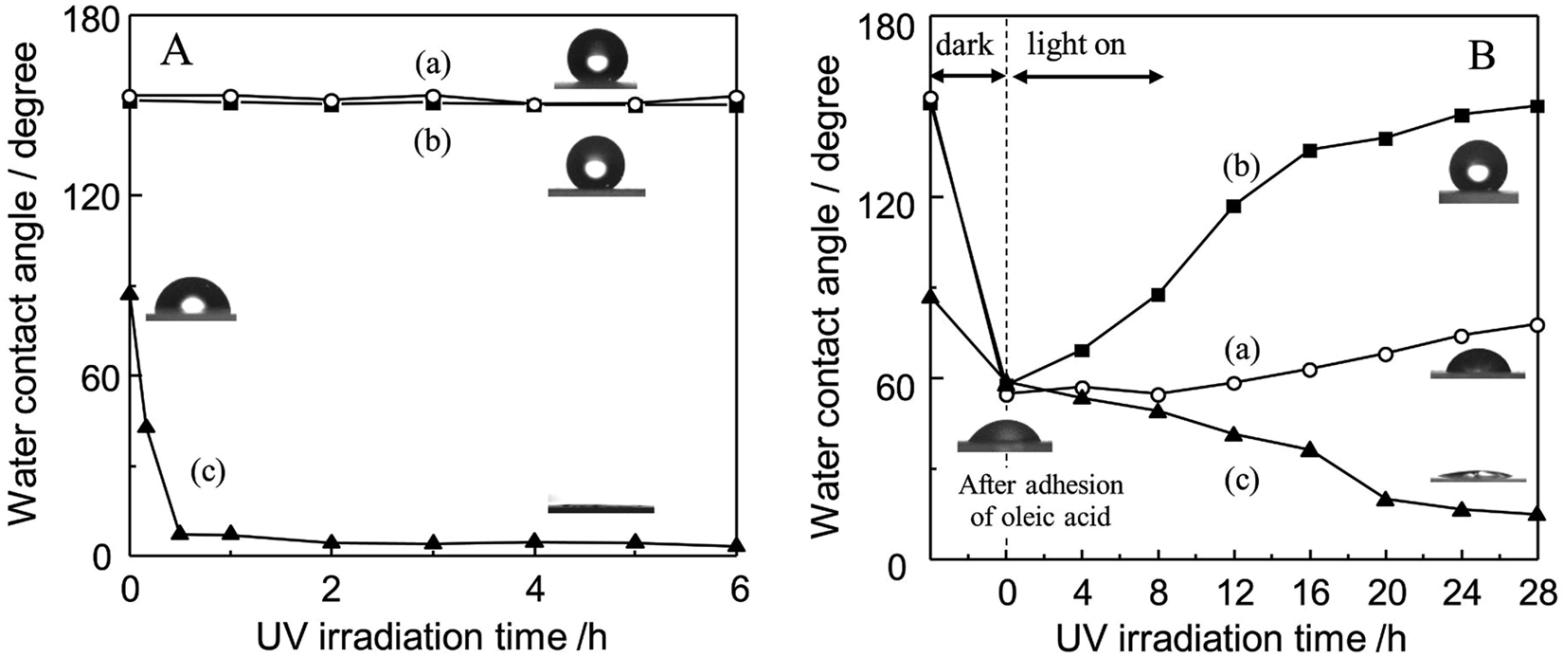
(**A**) Changes in the water contact angle under UV light irradiation and (**B**) water contact angle changes by adhesion of oleic acid on sample surface and following UV light irradiation (samples: (a) PTFE_*(25,400)*_/Q, (b) TiO_2_-PTFE_(*25*,*400*)_/Q, and (c) TiO_2_/Q). Inset of this figure shows the photographic images of water droplet on each sample.

The water contact angle measurements were carried out to evaluate the surface wetting properties of each sample. The water contact angle on PTFE_(*25*,*400*)_/Q and TiO_2_-PTFE_(*25*,*400*)_/Q was 153° and 152°, respectively, which fulfilled definition of superhydrophobic surface. The mixing of the small amount of TiO_2_ in the PTFE matrix was scarcely affect to the water repellent property. The water contact angle on conventional TiO_2_ thin film (TiO_2_/Q) was ca. 87°. As shown in Fig. 6(A), photoinduced superhydrophilicity was only observed in TiO_2_/Q under UV light irradiation. The water contact angle was gradually decreased to almost 0° after a short period of UV light irradiation time. Water droplet was completely spread on this surface. On the other hand, PTFE_(*25*,*400*)_/Q and TiO_2_-PTFE_(*25*,*400*)_/Q maintained almost the same water contact angle even after UV light irradiation. PTFE was a stable in the present experimental conditions. The mixing of the small amount of TiO_2_ in the PTFE matrix, which severally possesses the hydrophilic or hydrophobic natures, effectively suppress the spreading of water droplet on surface under UV light irradiation.

To evaluate the self-cleaning property by uses of photocatalytic performance of TiO_2_, the adhesion of oleic acid as a model of organic contaminants and its photocatalytic decomposition was investigated while measuring the water contact angle on each sample surface. As shown in Fig. 6(B), water contact angle changed to around 60° after adhesion of oleic acid on surface. In the FT-IR measurement, two typical peaks attributed to the CH stretching vibration of alkyl groups in the adhered oleic acid were observed at 2925 and 2855 cm^−1^ ^50^. Superhydrophobicity of PTFE_(*25*,*400*)_/Q and TiO_2_-PTFE_(*25*,*400*)_/Q was disappeared by covering the oleic acid on the top surface of these samples. The water contact angle on PTFE_(*25*,*400*)_/Q was not dramatically altered by UV light irradiation. The gradual decreases of water contact angle on TiO_2_/Q was observed by the photocatalytic removal of the adhered oleic acid and surface wettability changes due to the photoinduced superhydrophilicity. In contrast to these samples, TiO_2_-PTFE_(*25*,*400*)_/Q exhibited a unique surface wettability changes. Superhydrophobicity on this surface was recovered through the gradual increases of water contact angle under UV light irradiation. Roundish water droplet was finally observed by restoring its original surface state. The small amount of TiO_2_ mixed in the PTFE matrix was effectively worked as a photocatalyst for removal of the adhered oleic acid on surface, which realized a self-cleaning property for keeping a clean surface with superhydrophobicity.

In conclusion, surface coatings on a flat quartz substrate by periodically structured PTFE and TiO_2_-PTFE composite pillars were successfully achieved using a radio frequency-magnetron sputtering deposition technique through a stainless steel knitted wire net. By changing the size and height of pillars, roundish water droplet was clearly observed on the sample surface due to the surface characteristics change to the superhydrophobic state. The photocatalytic property of TiO_2_ was also imparted through the mixing of the small amount of TiO_2_ in PTFE pillar matrix by simultaneous deposition of two components. With keeping a balance of hydrophobic domain of PTFE and other domain consists of hydrophilized TiO_2_ under irradiation of UV light, this mixing technique realized a superhydrophobic surface possessing efficient photocatalytic self-cleaning effect. Such functions might be useful for maintenance free superhydrophobic coatings on various materials.

## Methods

### Preparation of samples

A radio frequency-magnetron sputtering deposition equipment was used for deposition of pure polytetrafluoroethylene (PTFE), polychlorotrifluoroethylene (PCTFE), fluorinated ethylene-propylene (FEP), TiO_2_ photocatalyst and composite of PTFE and TiO_2_. The base pressure in the chamber was set below 5 × 10^−4^ Pa, and the working pressure was adjusted to 1.0 Pa. The substrate was placed parallel to the sputtering target surface with substrate-target distance of ca. 50 mm. The depositions were performed at 298 K using a radio frequency power of 20 W. A flat quartz glass substrate (10 × 10 mm) was preliminarily washed in acetone, ethanol, and distilled water using an ultrasonic bath, then dried at 373 K for 1 h in an oven. Commercially available PTFE plate and anatase-type TiO_2_ (Ishihara Sangyo, Ltd., ST-01) pellet (diameter: 25.4 mm, thickness, 30 mm) were used as a sputtering target, respectively. In the co-deposition process, quadrant shape of PTFE plate and TiO_2_ pellet was combined with each other and used for a sputtering target. For a precise controlling of surface architecture on a flat quartz glass substrate, deposition was carried out through a stainless steel knitted wire net with a different size of square openings (100, 200, 400 and 635 mesh) for making a periodically structured PTFE pillars (Fig. 1). Sample names with pillars were denoted as PTFE_(*h*,*m*)_/Q (pillar height: *h* = ca. 3–40 μm, *m*: mesh size of wire net). PCTFE and FEP deposited samples were also prepared in the same manner.

### Characterization techniques

The surface architecture of samples was observed by optical and laser scanning microscope (KEYENCE, VK-9700). FT-IR spectra were recorded with a resolution of 4 cm^−1^ using a JASCO FT/IR-6100. X-ray photoelectron spectroscopy (XPS) measurements were carried out by using a Shimadzu ESCA-3200 using Mg Kα radiation. Ti K-edge X-ray absorption fine structure (XAFS) spectra were recorded at 298 K in the fluorescence mode at the BL-9A facility of the Photon Factory at the high energy acceleration research organization (KEK) in Tsukuba, Japan. The synchrotron radiation from a 2.5 GeV electron storage ring was monochromatized by Si(111) double crystals.

### Water contact angle measurement

Surface wettability was evaluated by the contact angle of a pure water droplet (ca. 2 μl) using a contact angle meter (Kyowa Interface Science Co., Ltd., DropMaster 300). The water contact angle measurement was performed at three different points on the film surface, and an average value was adopted. Surface wetting behavior was analyzed by using two models established by Wenzel as well as Cassie and Baxter^1,2^. The Wenzel equation (Eq. (1)) related to the homogeneous wetting regime is as follows:

1 $$\cos \,{\theta }_{W}=r\,\cos \,\theta $$

where *θ*_*W*_ is Wenzel apparent contact angle, *θ* is equilibrium contact angle on a smooth surface of same material and *r* is the surface roughness defined as a ratio of the true area of the solid surface to its nominal area. The Cassie-Baxter equation (Eq. (2)) postulated heterogeneous wetting regime is as follows:

2 $$\cos \,{\theta }_{CB}={\Phi }\,(\cos \,\theta +1)-1$$

where *θ*_*CB*_ is Cassie-Baxter apparent contact angle, *Φ* is the fraction of the solid in contact with the liquid. For evaluation of the self-cleaning property, contamination of each film surface by adhesion of oleic acid was performed by using an acetone solution of oleic acid (10 mmol/l, ca. 50 μl). UV light irradiation was carried out with a 200 W mercury xenon lamp (San-ei Electric Co., Ltd., UVF-204S) under controlled light intensity (Intensity at 360 nm: 5 mW/cm^2^).

### Data Availability Statement

All datasets generated during the performance of this study are available from the corresponding author upon request.

## Electronic supplementary material

Supplementary information
