# Supplementary material for: Multifunctional surface designed by nanocomposite coating of polytetrafluoroethylene and TiO2 photocatalyst: self-cleaning and superhydrophobicity
Source: Sci Rep. 2017 Oct 19;7:13628. doi: 10.1038/s41598-017-14058-9 (PMC5648811; doi:10.1038/s41598-017-14058-9)
Supplement: Supplementary file 1 — Supplementary information [file 41598_2017_14058_MOESM1_ESM.doc]

Supplementary information:

**Multifunctional surface designed by nanocomposite coating of polytetrafluoroethylene and TiO_2_ photocatalyst: self-cleaning and superhydrophobicity**

Takashi Kamegawa^1,2,*^, Koichi Irikawa^1^, and Hiromi Yamashita^1,3,*^

^1^Division of Materials and Manufacturing Science, Graduate School of Engineering, Osaka University, 2-1 Yamadaoka, Suita, Osaka 565-0871, Japan.

^2^NanoSquare Research Institute, Osaka Prefecture University, 1-2 Gakuencho, Nakaku, Sakai, Osaka 599-8570, Japan.

^3^Unit of Elements Strategy Initiative for Catalysts and Batteries (ESICB), Kyoto University, Katsura, Kyoto 615-8510, Japan.

Correspondence and requests for materials should be addressed to T.K. (e-mail t-kamegawa@21c.osakafu-u.ac.jp) or to H.Y. email (yamashita@mat.eng.osaka-u.ac.jp).


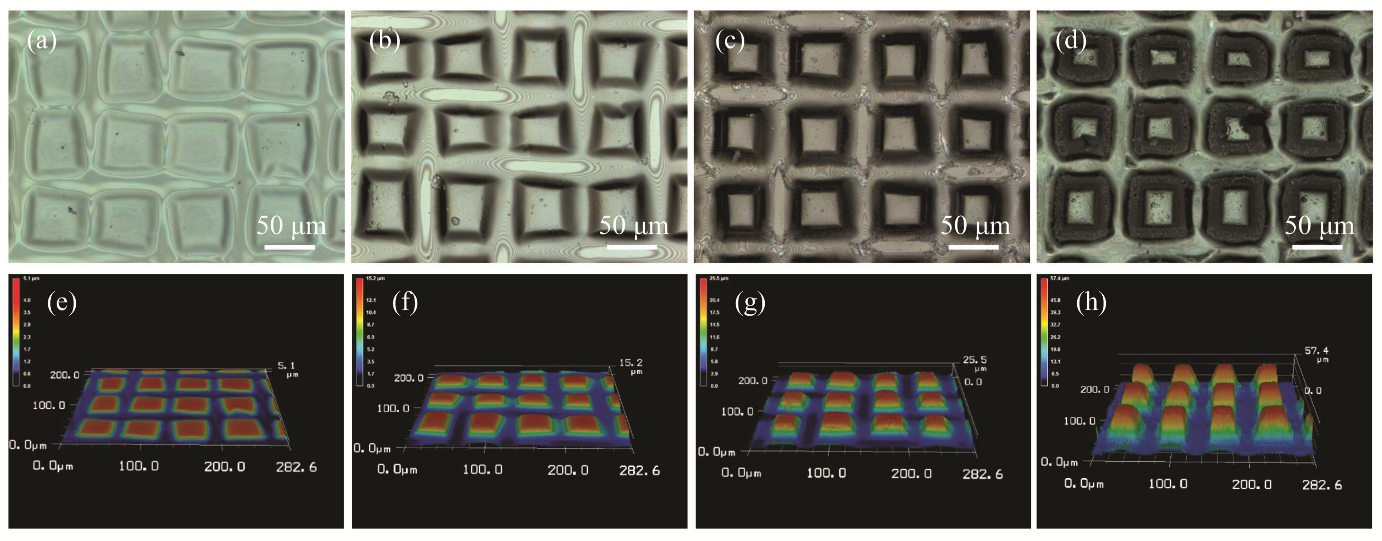


**Supplementary Figure 1.** (a-d) Optical microscope images and (e-h) 3D analytical laser microscope images of PTFE(*_h_*,*_400_*)/Q (*h* = ca. (a,e) 3, (b,f) 10, (c,g) 18, and (d,h) 40 μm).


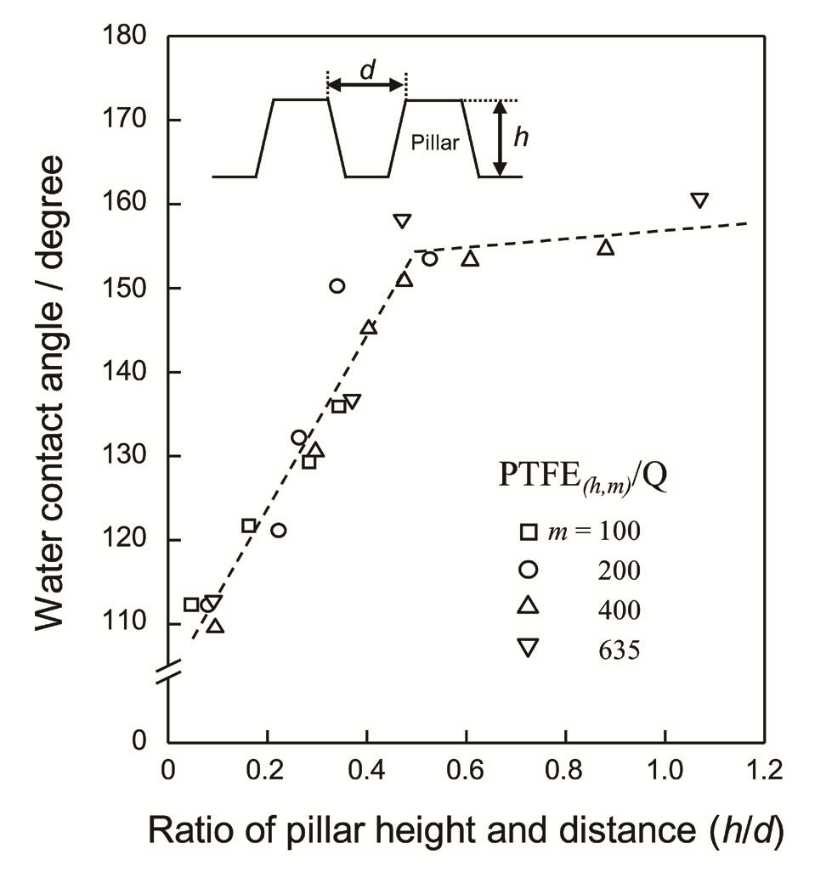


**Supplementary Figure 2.** Relationships between the ratio of pillar height to distance (*h/d*) and water contact angle on PTFE(*_h_*,*_m_*)/Q. Inset of this figure shows the cross-sectional views of a roughness geometry of pillars.


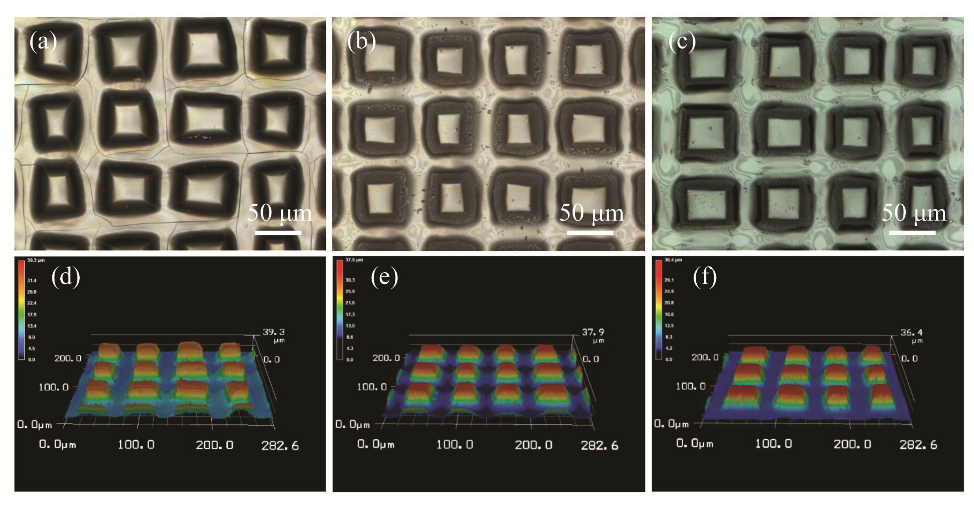


**Supplementary Figure 3.** (a-c) Optical microscope images and (d-f) 3D analytical laser microscope images of samples ((a,d) PCTFE(*_25_*,*_400_*)/Q, (b,e) FEP(*_25_*,*_400_*)/Q, and (c,f) PTFE*_(25,400)_*/Q).
